# Differential effects of ERK and p38 signaling in BMP-2 stimulated hypertrophy of cultured chick sternal chondrocytes

**DOI:** 10.1186/1478-811X-3-3

**Published:** 2005-02-03

**Authors:** Gwendolen C Reilly, Eleanor B Golden, Giovi Grasso-Knight, Phoebe S Leboy

**Affiliations:** 1 Department of Engineering Materials, Sir Robert Hadfield Building, University of Sheffield, Sheffield, S1 3JD, UK; 2 Biochemistry Department, School of Dental Medicine, University of Pennsylvania, 4001 Spruce Street, PA 19104-6003, USA

## Abstract

**Background:**

During endochondral bone formation, the hypertrophy of chondrocytes is accompanied by selective expression of several genes including type X collagen and alkaline phosphatase. This expression is stimulated by inducers including BMPs and ascorbate. A 316 base pair region of the type X collagen (Col X) promoter has been previously characterized as the site required for BMP regulation. The intent of this study was to examine the role of Mitogen Activated Protein (MAP) and related kinase pathways in the regulation of Col X transcription and alkaline phosphatase activity in pre-hypertrophic chick chondrocytes.

**Results:**

Using a luciferase reporter regulated by the BMP-responsive region of the type X collagen promoter, we show that promoter activity is increased by inhibition of extra-cellular signal regulated kinases 1 or 2 (ERK1/2). In contrast the ability of BMP-2 to induce alkaline phosphatase activity is little affected by ERK1/2 inhibition. The previously demonstrated stimulatory affect of p38 on Col X was shown to act specifically at the BMP responsive region of the promoter. The inhibitory effect of the ERK1/2 pathway and stimulatory effect of the p38 pathway on the Col X promoter were confirmed by the use of mutant kinases. Inhibition of upstream kinases: protein kinase C (PKC) and phosphatidylinositol 3-(PI3) kinase pathways increased basal Col X activity but had no effect on the BMP-2 induced increase. In contrast, ascorbate had no effect on the BMP-2 responsive region of the Col X promoter nor did it alter the increase in promoter activity induced by ERK1/2 inhibition. The previously shown increase in alkaline phosphatase activity induced by ascorbate was not affected by any kinase inhibitors examined. However some reduction in the alkaline phosphatase activity induced by the combination of BMP-2 and ascorbate was observed with ERK1/2 inhibition.

**Conclusion:**

Our results demonstrate that ERK1/2 plays a negative role while p38 plays a positive role in the BMP-2 activated transcription of type X collagen. This regulation occurs specifically at the BMP-2 responsive promoter region of Col X. Ascorbate does not modulate Col X at this region indicating that BMP-2 and ascorbate exert their action on chondrocyte hypertrophy via different transcriptional pathways. MAP kinases seem to have only a modest effect on alkaline phosphatase when activity is induced by the combination of both BMP-2 and ascorbate.

## Background

During skeletal development and growth, bone formation occurs either by intramembraneous or endochondral bone formation. In endochondral bone formation, which occurs at the growth plates of long bones, cartilage is formed first, then the chondrocytes undergo a proliferative phase followed by hypertrophy, changes in gene expression, and matrix calcification, after which the cartilage is replaced by bone. Although generally referred to as chondrocyte hypertrophy, cell enlargement is just one manifestation of the more complex process of chondrocyte maturation, which can be considered an end-stage of chondrocyte differentiation. It is important to define the mechanisms that induce chondrocyte maturation, not only to understand bone development, but also to help prevent hypertrophy and ossification during cartilage tissue engineering.

Hypertrophic chondrocytes are characterized by their increased levels of alkaline phosphatase (ALP), reduced levels of type II and IX collagens, and the emergence of type X collagen (Col X), which is a specific marker of hypertrophy [1,2]. Ascorbate and bone morphogenetic proteins (BMPs) are among the factors previously shown to be inducers of ALP gene expression in chondrocytes. Either of these inducers alone will elevate ALP activity in chondrocytes derived from pre-hypertrophic regions of avian cartilage, but the combined effect of BMP and ascorbate is more than additive [3]. In early studies with avian chondrocytes, ascorbate-induced increases in type X collagen expression appeared to parallel increasing alkaline phosphatase activity, suggesting that both Col X and ALP might be controlled by common pathways [4]. However, analyses of BMP-stimulated hypertrophy suggested that ALP activity gradually increased over a 3 day period, while Col X mRNA reached maximal levels within 24 h. Experiments in which pre-hypertrophic chick chondrocytes were transfected with luciferase constructs regulated by sequences from the avian type X collagen gene demonstrated that a "b2" region 2.6-2.0 kilobases upstream of the ColX transcription start site, when joined to 640 base pair (bp) region of the proximal promoter, was transcriptionally activated by BMP-2, -4, and -7 [5]. Northern blot analyses after cyclohexamide treatment showed that new protein synthesis is not required for BMP-induced Col X expression [3]. Additional studies indicated that the mechanism for type X collagen promoter regulation probably involves BMP-activated Smads interacting with a Runx2/Cbfa1 transcription factor [6], and that retinoic acid stimulation of Col X expression is via the same 316 bp region [7,8]. Although long-term (4–7 day) treatment of chondrocytes with ascorbate results in increased levels of type X collagen mRNA [9], there is no data concerning the ability of ascorbate to regulate the type X collagen promoter.

In osteoblastic cells, BMPs and ascorbate have been shown to operate via mechanisms that at least partly involve mitogen activated protein kinases (MAP kinases). For example, ascorbate promotes extracellular matrix production which, in turn, activates the extracellular-signal regulated kinases, (ERK1/2 or p42/ p44) in an osteoblastic cell line [10]. MAP kinases including ERK1/2 [11,12], p38 [13] and PI3 (phosphatidylinositol 3-) kinase [14] have also been reported to be required for BMP-dependent induction of osteoblast differentiation. However, these pathways can act oppositely in certain BMP induced processes such as osteocalcin synthesis by osteoblasts [15].

In general, MAP kinase pathways involving ERK1/2 stimulate proliferation, growth and differentiation, whereas those that stimulate p38 kinase lead to differentiation and apoptosis [16]. In early stages of chondrocyte differentiation, an increase in p38 and decrease in ERK1/2 activity is required for the progression to cartilage nodule formation in chick limb buds [17]. In hypertrophying chondrocytes p38 has been shown to be required for Col X mRNA synthesis [18]. In apoptosis of articular chondrocytes [19] and other cell types [20], ERK1/2 inhibits and p38 stimulates the apoptotic pathway.

Chick sternal chondrocytes are a popular model for the study of chondrocyte maturation because under normal development chondrocytes from the cephalic portion of the sternum undergo hypertrophy followed by mineralization and bone formation, whereas the caudal portion remains as cartilage [3,8]. In this study we investigate the roles of ERK1/2, p38 and two upstream pathways, protein kinase C (PKC) and PI3 kinase, in the maturation of chick prehypertrophic sternal chondrocytes induced by BMP-2 and ascorbate.

## Results

### ERK signaling inhibits transcription of the BMP-2 responsive type X collagen promoter but is not involved in the regulation of alkaline phosphatase activity

**Figure 1:**
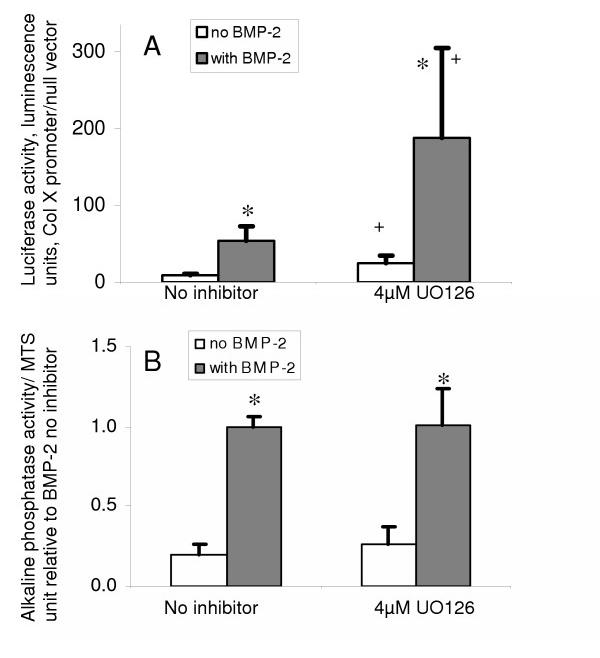
**Effects of BMP-2 and the ERK inhibitor, UO126, on the Col X promoter and alkaline phosphatase activity in chick cephalic sternal chondrocytes. A: **Activity of the b2/640 type X collagen promoter; 24 hrs after seeding cells were transfected with PGLb2/640 and pRLnull luciferase vectors, 5 hrs after transfection 4 μM U0126 or vehicle (DMSO) was added. BMP-2 was added to selected wells after a further hour. Values are mean ± S.D of the mean ratio of promoter to empty vector fluorescence units, for 6 experiments assayed in triplicate. **B: **Alkaline phosphatase activity; 24 hrs after seeding, medium was changed and 4 μM U0126 or vehicle was added. BMP-2 was added to selected wells after a further hour. Cell extracts were prepared 72 hrs later. Data was obtained using 5 different isolates of chondrocytes assayed in triplicate. Values are mean ± SEM of 12–15 samples normalized to within experiment controls treated with BMP-2 but no inducers, *:p < 0.01 group differs from non BMP-2 treated group within inhibitor treatment, +: p < 0.05 that group differs from group with no UO126.

Studies with the ERK1/2 inhibitor U0126 indicated that blocking ERK1/2 signaling increased the activity of the type X collagen promoter but had no effect on alkaline phosphatase (ALP) activity in chick cephalic sternal chondrocytes (Fig. 1). Cells transfected with luciferase reporter plasmid containing the BMP-responsive b2/640 region of the Col X promoter showed a 3-fold (p < 0.05) increase in luciferase expression, as a ratio to the pRL null control vector, after the addition of 4 μM U0126, both with and without exogenous BMP-2 (Fig 1A). In contrast neither basal nor BMP-stimulated ALP activity were significantly changed in the presence of U0126 (Fig. 1B).

ALP activity was highly variable between cell isolates and is expressed here normalized to BMP-2 treated controls for the purpose of combining experiments, typical ALP values ranged from approximately 0.5–2 nmol/min/μg DNA in controls to between 4 and 12 nmol/min/μg DNA in BMP-2 treated cultures.

**Figure 2:**
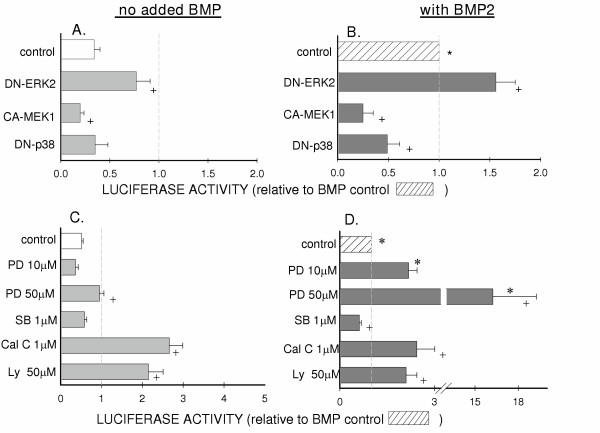
**Effects of MAP kinase manipulation on b2/640 type X collagen promoter activity, in chick cephalic sternum chondrocytes. A **and **B**: Mutant kinases; 24 hrs after seeding, cells were transfected with luciferase vectors and 0.5–1 μg mutant kinase DNA. After 5 hrs medium was changed (**A**) or medium changed and BMP-2 added (**B**). **C **and **D**: Inhibitor treatments; 5 hours after transfection with luciferase vectors, medium was changed and kinase inhibitor or vehicle (DMSO) was added as indicated. In **D **30 ng/ml BMP-2 was added one hr after medium change. Data obtained using at least 2 independent isolates of chondrocytes, assayed in triplicate. Values are mean ± SEM of luciferase ratios of type X collagen promoter activity to control vector, normalized to BMP-2 treated controls. *:p < 0.05 that luciferase ratio differs from non BMP-2 treated group, +: p < 0.05 that luciferase ratio differs from group with no MAP kinase manipulation.

The effects of altered ERK1/2 signaling on Col X promoter activity in chick sternal chondrocytes was further studied both by transfection with mutant kinases and by treatment with additional kinase inhibitors. Col X promoter activity was increased, both in the presence and absence of BMP-2, when the mitogen-stimulated ERK pathway was suppressed by transfecting chondrocytes with dominant negative ERK-2 (Figs. 2A, 2B). Conversely, stimulating the ERK1/2 pathway by over-expressing constitutively active MEK1, an upstream kinase of ERK1/2, decreased promoter activity by 50% (p < 0.05) and in BMP-2 treated cells it eliminated any BMP response.

As seen with the ERK1/2 inhibitor U0126, treatment with the more specific ERK1/2 inhibitor PD098059 increased b2/640 Col X promoter activity, in the presence of BMP-2 (Fig. 2D). Dose response experiments indicated that concentrations of PD98059 as low as 10 μM significantly increased luciferase expression 2-fold (p < 0.001) in BMP-treated cells, but not in the absence of BMP-2 (Fig. 2C). At a higher does, 50 μM, of PD90859 luciferase levels in BMP-2 treated cells were 10–20 fold higher (p < 0.005) than BMP-containing cultures without inhibitor (Fig. 2D), at this dose PD90859 also stimulated the promoter in the absence of BMP-2 (Fig. 2C).

### p38 MAP kinase signaling contributes to the response of the type X collagen promoter to BMP-2

Transfection with dominant negative p38 caused a decrease in Col X promoter activity in BMP-2 treated cephalic chondrocytes, reducing activity to half of that seen in BMP-2 treated controls (p < 0.005) and eliminating the BMP-2 response (Figs. 2A and 2B). Similarly, 1 μM SB 203580, an inhibitor of p38, significantly decreased BMP-stimulated promoter activity (Fig. 2D), but had little effect on promoter activity in the absence of BMP-2 (Fig. 2C).

### Inhibiting PKC and PI3 kinases increases type X collagen promoter activity

Addition of either PI3 kinase inhibitor or PKC inhibitor resulted in similar stimulation of the collagen type X promoter. Calphostin C, a PKC inhibitor, increased activity in BMP-2 treated cells more than 2-fold, an effect similar to that seen with the ERK1/2 inhibitor PD98059 at 10 μM (Fig. 2D). Similarly, 50 μM LY294002, a PI3 kinase inhibitor, stimulated the b2/640 promoter approximately 2-fold (Fig. 2D). However, both of these inhibitors also increased transcription of the collagen type X promoter in non-BMP-2 treated cells to levels as high as seen with the combination of BMP-2 and the respective inhibitor treatment (Fig. 2C).

### Kinase inhibitor effects on viable cell number

To assess the possible effects of protein kinase inhibitors on cell proliferation and survival, we measured relative numbers of live cells using a tetrazolium (MTS) assay. The results indicated that all cultures treated with inhibitors, with and without BMP-2 and/or ascorbate, had cell numbers within 10% of untreated controls (data not shown).

### Ascorbate has no effect on the type X collagen promoter and stimulates alkaline phosphatase activity regardless of kinase inhibitor treatment

**Figure 3:**
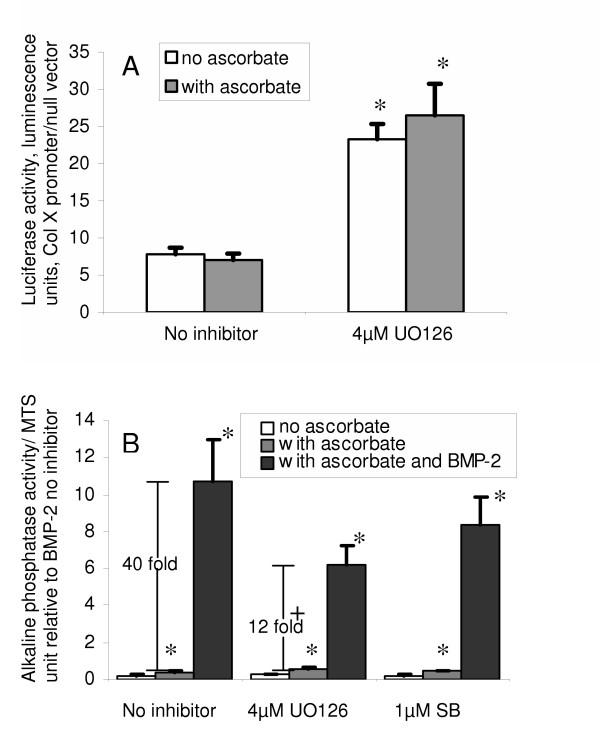
**Effects of ascorbate and MAP kinase inhibitors on the Col X promoter and alkaline phosphatase activity in chick cephalic sternal chondrocytes. A: **Activity of the b2/640 type X collagen promoter; 24 hrs after seeding cells were transfected with luciferase vectors, 5 hrs after transfection 4 μM U0126 or vehicle were added. Ascorbate was added to selected wells after a further hour. Values are mean ± S.D of the mean ratio of promoter to empty vector fluorescence units, for 3 experiments assayed in triplicate. **B: **Alkaline phosphatase activity; 24 hrs after seeding, medium was changed and 4 μM U0126 or vehicle was added. Ascorbate or ascorbate with BMP-2 was added to selected wells after a further hour. Cell extracts were prepared 72 hrs later. Data was obtained using 5 different isolates of chondrocytes assayed in triplicate. Values are mean ± SEM of 12–15 samples normalized to within experiment controls treated with BMP-2 but no inducers. *: p < 0.05 that group differs from non-supplemented group within inhibitor treatment. The increase in ALP caused by BMP-2 addition is shown, this increase is significantly smaller in UO126 treated cells, +:p < 0.05.

We examined the effect of 75 μM ascorbate-2-phosphate on the activity the Col X promoter in cultures treated with kinase inhibitors. Col X promoter activity was unaffected by addition of ascorbate, and 4 μM of the ERK1/2 inhibitor U0126 increased promoter activity to comparable levels both with and without ascorbate (Fig. 3A).

### The increase in alkaline phosphatase activity caused by adding BMP-2 to ascorbate treated cultures is reduced by ERK inhibitors

ALP activity in the absence of exogenous BMP was stimulated at least 2-fold in ascorbate-treated cultures without inhibitors, as previously reported, and this stimulation was not significantly affected by addition of either ERK1/2 or p38 inhibitors (Fig. 3B). In cultures treated with ascorbate and BMP-2 addition of ERK1/2 inhibitors resulted in ALP levels that were <60% of the level seen in cells without inhibitor (Fig. 3B). The increase caused by BMP-2 addition, relative to ascorbate only-treated cultures was significantly reduced by treatment with U0126 (p < 0.05). The p38 inhibitor SB203580 did not cause a statistically significant inhibition of alkaline phosphatase activity. PI3 kinase and PKC inhibitors had no significant effects on ALP activity (data not shown).

## Discussion

The present studies demonstrate that ERK1/2 inhibition increases activity of the BMP responsive region of the type X collagen promoter. This indicates that ERK1/2 signaling interferes with the ability of BMP-induced signals to stimulate type X collagen transcription. Interestingly ERK1/2 has also been shown to inhibit type I collagen expression in an osteoblastic cell line [21] suggesting there may be a common pattern of ERK1/2 inhibition of collagen transcription pathways.

In contrast to the stimulatory effects of inhibiting the ERK pathway, p38 inhibition blocked BMP-stimulated Col X promoter activity. Zhen et al. [18] and Beier and Luvalle [22] also showed that p38 signaling is important for regulation of Col X expression, Beier and Luvalle suggested that the proximal promoter contained a site for p38 action. Here, we have confirmed these results and narrowed the region of p38 responsiveness to within the region of the Col X promoter that is also BMP responsive.

While the classical pathway for BMP signaling is via activation of R-Smads, there is also evidence for BMP signaling via a TGF-activated kinase (TAK1) leading to p38 signaling [23-26]. However, in preliminary experiments Smad1 over-expression increased BMP-stimulated Col X promoter activity even in the presence of DN-TAK1 (data not shown). This suggests that BMP-activated Smad signaling and not TAK1 signaling is the major factor in Col X promoter regulation. Taken together these data suggest that the role of p38 is as a co-activator of Smads or Runx-2 rather than a downstream effector of BMP signaling.

Inhibiting either protein kinase C (PKC) or phosphatidylinositol 3-kinase (PI3 kinase) increased type X collagen promoter activity both in BMP-2 treated cultures and controls. Both PKC and PI3 kinase have been reported to negatively regulate p38 [18] and positively regulate ERK1/2 [20,27,28]. The complex effects in which these kinases stimulate basal Col X promoter activity but inhibit BMP-2 from stimulating additional activity may be due to their simultaneously affecting both of these pathways.

Although alkaline phosphatase expression, like type X collagen expression, increases during chondrocyte hypertrophy and is stimulated by BMPs, its regulation clearly differs from Col X in several respects. ERK1/2 inhibition has little effect on the ALP activity induced by BMP-2 or ascorbate acting alone and reduces the ability of BMP-2 to further stimulate ALP activity in ascorbate treated cultures. Inhibiting p38 does not have a clear effect on BMP-stimulated alkaline phosphatase activity in this model although it has been shown to reduce ALP activity in long-term (12–15 day) micromass cultures [29].

Preliminary studies with cyclohexamide indicate that new protein synthesis is required for the up-regulation of alkaline phosphatase mRNA in response to BMP-2. We propose that these differences reflect a direct Smad-mediated effect of BMPs on type X collagen expression and an indirect effect on ALP expression.

**Figure 4:**
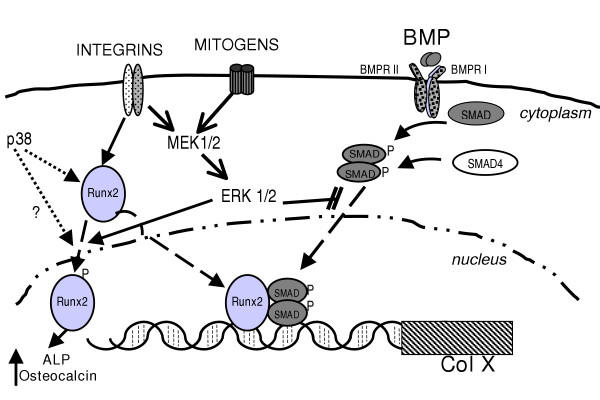
**Possible mechanism for kinase involvement in BMP stimulated type X collagen expression. **ERK1/2 is proposed to suppress activated Smad translocation to the nucleus as described by Kretschmer et al. [30] and thereby inhibit Col X transcription. ERK-specific Runx2 phosphorylation is required for regulation of typical osteoblastic genes but may not be required for Col X expression. However p38 facilitates Col X transcription, possibly via Runx2 phosphorylation and although was not shown to be involved in short term increases in ALP in our model, has been shown to be involved in long term increases in ALP over 15 days of chondrocyte differentiation in culture (question mark) [29].

A mechanism which could account for the observed effects of ERK and p38 signaling on expression of type X collagen and ALP is presented in Fig. 4. The simplest explanation for our observation that a decrease in ERK1/2 signaling causes increased type X collagen promoter activity is that ERK1/2 can phosphorylate the linker region of BMP-activated Smads, preventing nuclear translocation of activated Smads, as suggested by Kretzschmar et al. [30]. Alternatively, products of ERK1/2 signaling may act directly on a silencer within the type X collagen promoter such as the region identified by Beier et al. [31] at 2864-2410 base pairs which would overlap with our b2-containing construct (2649 -2007).

Evidence that BMP stimulation of type X collagen requires both activated R-Smads and Runx2 has been previously reported [5,7]. Little is known concerning kinase phosphorylation of Runx2, except for the report that ERK phosphorylates Runx2 and increases its binding to the osteocalcin promoter [32] in osteoblasts. If ERK phosphorylation of Runx2 were required for BMP-stimulated type X collagen transcription, we might expect ERK1/2 inhibition to decrease the activity of the Col X promoter. However, as ERK1/2 inhibition increases Col X promoter activity while partially inhibiting ALP we propose that the Runx2-Smad complex binding to the Col X promoter may not be phosphorylated by ERK1/2, but that ALP expression does require Runx2 phosphorylated by ERK1/2.

As well as its demonstrated role in Col X expression as found here and by [18,31], there are also reports that p38 inhibitors block osteoblast differentiation [33,34]. Because Runx2 plays an important role in both osteogenesis and chondrocyte maturation, we have suggested that p38 may function in Runx2 expression, activation or nuclear translocation. However, there are many other possible roles, including the suggestion that p38 is downstream of BMP-activated Smad signaling [35].

Retinoic acid [7,8], another stimulator of chondrocyte hypertrophy has also been shown to act at the BMP-2 responsive b2 region of the type X collagen promoter, however ascorbate, which does produce an increase in type X collagen mRNA expression [4], does not seem to have any effect on this promoter region. These results, in combination with previous findings that Col X mRNA expression only occurs after 4–9 days stimulation with ascorbate [9], suggest that the effects of ascorbate on regulation of type X collagen expression are via a separate mechanism than BMP stimulation and are probably indirect.

## Conclusions

Elucidating the signaling pathways by which chondrocytes are driven to hypertrophy is necessary in order to better understand skeletal development, cartilage disease and improve the design of tissue engineered cartilage. We showed here that the ERK1/2 pathway inhibits type X collagen production by either directly or indirectly acting at the BMP responsive region of the promoter. p38 kinase signaling stimulates type X collagen transcription at the same promoter region, probably in conjunction with BMP-2 activated Smads. The factor upstream of p38 in this stimulatory pathway is unknown. Alkaline phosphatase activity is likely to be regulated in a different way from type X collagen since MAP kinases do not contribute in the same way to this pathway. Although ascorbate and BMPs both induce hypertrophy in chondrocytes ascorbate does not act at the same region of the Col X promoter as BMPs.

## Methods

### Inhibitors and plasmids

The ERK1/2 inhibitor PD98059, which blocks the upstream kinase (MEK1) of ERK1/2, the p38 inhibitor SB203580, and the PKC inhibitor Calphostin C were obtained from Sigma (St. Louis, MO). UO126, also a MEK1 inhibitor, was obtained from Biomol (Plymouth Meeting, PA) and LY294002, a phosphatidylinositol 3- (PI3) kinase inhibitor from Cell Signaling Technology (Beverly, MA). Plasmids were kindly donated as follows: constitutively active MEK1 from Michael Webber (University of Virginia); dominant negative p38 [36] from Roger Davis at the Howard Hughes Medical Institute, University of Massachusetts Medical School, dominant negative ERK2 [37] from Melanie Cobb at University of Texas Southwestern Medical Center.

### Cell Culture

Chondrocytes were cultured as previously described [3]. Cephalic and caudal sternal chondrocytes were isolated from 15 day chick embryos and cultured for 5 days. Dissection of chick cartilage was performed under a University of Pennsylvania IACUC exemption. On day 5 non-adherent cells were removed and plated in 12 well plates at 300,000 cells /well in DMEM supplemented with 10% NuSerum, 2 mM L-glutamine, 100 U/ml penn/strep and 4 U/ml hyaluronidase, to promote cell attachment.

### Transfection of cephalic (pre-hypertrophic) sternal chondrocytes

On day 1 of secondary culture (24 hrs after plating) the cell layer was washed in HBSS and the media changed to DMEM supplemented with 10% FBS in place of NuSerum. Cells were co-transfected with pGL2 plasmid containing the b2/640 type X collagen promoter region attached to a firefly luciferase reporter (0.2–1 μg/well) (Promega, Madison, WI) and pRL null plasmid attached to a *renilla *luciferase reporter (0.4 μg/well) (Promega) which served as a transfection control [5]. When appropriate, mutant plasmids were added at 0.5 or 1 μg/well along with the luciferase vectors. Luciferase and mutant kinase plasmids were transfected either using CaPO_4 _precipitation or Fugene transfection reagent at 6 μl/ml (Promega). Since preliminary experiments using green fluorescent protein showed that Fugene was more effective in terms of numbers of cells transfected, this method was used for the majority of the experiments; however, relative outcomes between controls and treated cells were not affected by the transfection method.

Transfection proceeded for 5 hrs after which the cell layer was rinsed twice in HBSS and cultured with serum free medium (DMEM with 2 mM L-glutamine, 100 U/ml penn/strep, 4 U/ml hyaluronidase, 60 ng/ml insulin, 1 mM cysteine, and 10 pM triiodothyronine). Some wells were supplemented with 75 μM ascorbate-2-phosphate (Wako, Takara, Japan) or 30 ng/ml human recombinant BMP-2 (Wyeth, Cambridge, MA). Where inhibitors were used they were added at this point and cells incubated for 1 hr before the addition of BMP-2. Cells were cultured for a further 48 hours, then lysed and assayed using a dual luciferase assay kit (Promega).

### Alkaline Phosphatase Assay

For alkaline phosphatase assays, cells were switched to serum free medium on day 1 of secondary culture, inhibitors were added and cells incubated for 1 hr before the addition of ascorbate or BMP-2, as described for the luciferase assay. Cells were cultured for a further 72 hrs and then rinsed twice in HBSS. Cells numbers were assayed either by DNA quantification (CyQUANT cell proliferation assay kit, Molecular Probes, Eugene, OR) or by MTS tetrazolium salt assay of mitochondrial activity (Cell Titer 96 AQueous One Solution Cell Proliferation Assay, Promega). When MTS was used, a 1:10 dilution of MTS was applied in phenol red-free media for 30–60 minutes, 200 μl of media plus MTS was transferred to a 96 well plate and assayed in a 'Multiskan ascent' plate reader (Thermolabsystems, Franklin, MA). The cell layer was then washed twice in HBSS and extracted with 0.15 M Tris, pH 9 with 0.1 mM ZnCl_2_, 0.1 mM MgCl_2 _and 1% Triton X-100 for 30 mins at 37°C, followed by overnight storage at 4°C. A sample of the cell lysate was reacted with p-nitrophenyl phosphate substrate in 1.5 M Tris buffer pH 9 with 1 mM ZnCl_2 _and 1 mM MgCl_2_. Phosphatase activity was measured specrophotometrically at 410 nm with 1 absorbance unit equivalent to 64 nmol of product. For DNA analysis, cells were trypsinized and a subsample of cell suspension centrifuged, the cell pellet lysed with the CyQUANT lysis buffer and the fluorescent DNA dye added. The resulting solution was transferred to a 96 well plate and DNA assayed fluorometrically. The remaining cells were extracted for the alkaline phosphatase assay as above. Alkaline phosphatase enzyme levels were calculated as nmol p-nitrophenol product per minute normalized to MTS units or μg DNA.

### Statistical analysis

Statistics were performed using Minitab™ software. After expressing results as a ratio of experimental/control within each experiment, the data from at least 3 experiments were combined. The combined data was tested for normality using the Anderson-Darling test. Normally distributed or non-parametric data were tested for differences between treatments using two-sample Students t-test or the Mann-Whitney test respectively. Where groups of experiment means were compared a paired t-test was used.

## List of Abbreviations

ALP – Alkaline Phosphatase

BMP – Bone Morphogenetic Protein

bp – base pairs

CA – constitutively active

cbfa – core binding factor alpha 1

Col X – Collagen type X

DN – dominant negative

ERK – extracellular signal regulated protein kinase

MAP – Mitogen activated protein

MEK – ERK kinase

MTS – 3-(4,5-dimethylthiazol-2-yl)-5-(3-carboxymethoxyphenyl)-2-(4-sulfophenyl)-2H-tetrazolium, inner salt

PI3 – phosphhatidylinositol 3

PKC – Protein Kinase C

Runx – Runt associated protein

TAK – Transforming growth factor beta activated kinase

## Competing interests

The author(s) declare that they have no competing interests.

## Authors' contributions

GCR was involved in experimental design, performed 90% of the experiments, analyzed the data and wrote the first draft of the manuscript. EBG isolated chondrocytes, performed preliminary experiments and contributed to trouble-shooting of the methods. GG-K, performed preliminary experiments and developed methodology. PSL was involved in experimental design and data analysis, wrote portions of and edited the whole manuscript and was the project supervisor.
